# SrcA is a chaperone for the *Salmonella* SPI-2 type three secretion system effector SteD

**DOI:** 10.1099/mic.0.000732

**Published:** 2018-11-20

**Authors:** Camilla Godiee, Ondrej Cerny, Charlotte H. Durkin, David W. Hoiden

**Affiliations:** MRC Centre for Molecular Bacteriology and Infection, Imperial College London, London, SW7 2AZ, UK

**Keywords:** *Salmonella*;, type III secretion system, effector, chaperone

## Abstract

Effector proteins of type three secretion systems (T3SS) often require cytosolic chaperones for their stabilization, to interact with the secretion machinery and to enable effector delivery into host cells. We found that deletion of *srcA*, previously shown to encode a chaperone for the *Salmonella* pathogenicity island 2 (SPI-2) T3SS effectors SseL and PipB2, prevented the reduction of mature Major Histocompatibility Complex class II (mMHCII) from the surface of antigen-presenting cells during *Salmonella* infection. This activity was shown previously to be caused by the SPI-2 T3SS effector SteD. Since *srcA* and *steD* are located in the same operon on the *Salmonella* chromosome, this suggested that the *srcA* phenotype might be due to an indirect effect on SteD. We found that SrcA is not translocated by the SPI-2 T3SS but interacts directly and forms a stable complex with SteD in bacteria with a 2 : 1 stoichiometry. We found that SrcA was not required for SPI-2 T3SS-dependent, neutral pH-induced secretion of either SseL or PipB2 but was essential for secretion of SteD. SrcA therefore functions as a chaperone for SteD, explaining its requirement for the reduction in surface levels of mMHCII.

## Abbreviations

ACN: acetonitrile
LB: Luria-Bertani
mMHCII: mature Major Histocompatibility Complex class II
NiNTA: nickel-nitrilotriacetic acid
p.i.: post-invasion
pI: isoelectric point
PNS: post-nuclear supernatant
SEC-MALS: size exclusion chromatography-multiangle light scattering
SPI-2: *Salmonella* pathogenicity island 2
T3SS: type three secretion system
RT-PCR: reverse transcription polymerase chain reaction

## Introduction

Non-flagellar type three secretion systems (T3SS) of Gram-negative bacterial pathogens are needle-like surface structures that deliver virulence proteins (effectors) into eukaryotic cells [1]. Several T3SS proteins interact with chaperones in the bacterial cell. At least three chaperone classes exist: class I chaperones bind effectors, class II chaperones interact with translocon proteins (secreted proteins required for effector delivery across the eukaryotic membrane) and class III chaperones bind to needle subunits. Class I chaperones can be further subdivided into either class IA chaperones, which are specific for a single effector and are usually encoded in close proximity to the gene for their associated effector, or class IB chaperones, which act more broadly on several effectors and are usually encoded within the operon of the T3SS machinery [2, 3]. Class I chaperones are normally small proteins with an acidic iso-electric point (pI) that bind and stabilize effectors, holding them in a secretion-competent state, presumably through partial unfolding, while the chaperone remains in the bacterial cell [3, 4]. In the absence of its chaperone, delivery of the associated effector through the secretion system is either abolished or greatly reduced [5–7].

Serovars of *Salmonella enterica* are intracellular pathogens of mammals whose virulence relies on the delivery of approximately 30 effectors into host cells through the *Salmonella* pathogenicity island 2 (SPI-2) T3SS [8]. Infection of dendritic cells by *Salmonella enterica* serovar Typhimurium results in a SPI-2 T3SS-dependent decrease in surface levels of mature Major Histocompatibility Complex class II (mMHCII), which prevents antigen presentation and T-cell activation, a key requirement for the development of adaptive immunity to *Salmonella* [9, 10]. This activity results from the ubiquitination and lysosomal degradation of mMHCII molecules and is induced by the SPI-2 effector SteD. SteD is an integral membrane protein of 111 residues and probably functions as an adaptor, recruiting a host cell E3 ligase to drive the ubiquitination of a lysine residue in the cytoplasmic tail of the *β* chain of MHCII [11]. After translocation, SteD integrates into membranes of the *trans*-Golgi network and intracellular vesicles containing mMHCII. SteD has two transmembrane regions and both its N and C termini protrude into the host cytoplasm [11].

Inspection of the *steD* locus revealed that it lies adjacent to *srcA*, whose protein has been characterized previously as a *Salmonella* T3SS chaperone for the SPI-2 T3SS effectors SseL and PipB2 [12]. In this study, we investigated if SrcA is a chaperone for SteD. We found that SrcA binds to SteD, maintains its stability in *Salmonella* cells and is required for its translocation into the host cell. However, SrcA was not required for neutral pH-induced secretion of SseL and PipB2. Our results therefore suggest that SrcA is a class IA chaperone for SteD.

## Methods

### Bacterial strains, plasmids and antibodies

*Salmonella enterica* serovar Typhimurium (14028s) wild-type and all mutant strains are listed in Table S1 (available in the online version of this article). Bacteria were grown in Luria-Bertani (LB) medium supplemented with carbenicil-lin (50 μg ml^−1^), kanamycin (50 μg ml^−1^) or chloramphenicol (30 μg ml^−1^) as appropriate. All plasmids used are listed in Table S2. All primary antibodies used are listed in Table S3.

### Cell culture and infection

Mel Juso cells were maintained in Dulbecco’s modified Eagle medium containing 10 % FCS (Sigma) at 37 °C in 5 % CO_2_. Mel Juso cells were infected for 30 min at a m.o.i. of 100 with late log-phase *Salmonella* grown in LB medium as described previously [11]. Cells were washed twice with PBS and incubated in fresh medium containing gentamicin (100 μg ml^−1^) for 1 h to kill extracellular bacteria. After 1 h, the antibiotic concentration was reduced to 20 μg ml^−1^, and the cells were processed 20 h post-invasion (p.i.).

### Flow cytometry

Surface levels of mMHCII were measured following infection of Mel Juso cells as described previously [11] with minor modifications. In brief, Mel Juso cells were detached using 2 mM EDTA in PBS 20 h p.i. All antibodies were diluted in FACS buffer (5 % FCS and 1 mM EDTA in PBS). See Table S3 for information on primary antibodies, secondary antibodies were purchased from Life Technologies, UK. Cells were labelled with mouse anti-mMHCII primary antibody (L243) at 1 : 300 for 30 min on ice, washed in cold PBS, then labelled with donkey anti-mouse secondary antibody at 1 : 300 for 30 min on ice. After washing with cold PBS, cells were fixed in 3.7 % paraformaldehyde for 1 h at room temperature and permeabilized with 0.1 % Triton X-100 in FACS buffer for 10 min at room temperature. Subsequently, infected cells were labelled with goat anti-*Salmonella* CSA-1 primary antibody at 1 : 500 for 30 min on ice, washed in cold PBS, then labelled with donkey anti-goat secondary antibody at 1 : 300 for 30 min on ice. Surface levels of mMHCII were calculated as median fluorescence of infected cells (CSA-1 positive)/median fluorescence of non-infected cells (CSA-1 negative) × 100. Data were acquired using a Fortessa flow cytometer (BD Biosciences) and analysed using FlowJo v10 software.

### Immunofluorescence microscopy

Cells were seeded onto coverslips and infected as described above. At 20 h p.i. cells were washed in PBS, fixed in 3 % paraformaldehyde in PBS for 15 min at room temperature, then the paraformaldehyde was quenched by incubation with 50 mM NH_4_Cl for 10 min. All antibodies were diluted in 10 % horse serum (Sigma) and 0.1 % saponin in PBS. Coverslips were washed in 0.1 % saponin in PBS then incubated with appropriate primary antibodies for 1 h at room temperature, washed in 0.1 % saponin in PBS, then incubated with secondary antibodies for 1 h at room temperature. Finally coverslips were incubated with DAPI for 5 min, washed in 0.1 % saponin in PBS then mounted onto glass slides using Aqua-Poly/Mount (Polysciences). See Table S3 for information on primary antibodies and dilutions used. Alexa Fluor-conjugated donkey anti-rat 488, anti-goat 647 and anti-mouse 555 were purchased from Life Technologies, UK. Coverslips were imaged using an LSM 710 inverted confocal laser-scanning microscope (Zeiss GmbH).

### In *vitro* pH shift protein secretion assay

An *in vitro* secretion assay was carried out as previously described [13]. Bacterial strains were grown overnight in LB medium with or without antibiotic, and subcultured 1 : 50 into 6 ml MgM-MES medium pH 5.0 for 4 h to induce the assembly and activation of the SPI-2 T3SS. The full cell culture was collected at room temperature by centrifugation, then re-suspended in MgM-MES at pH 7.2 and incubated for 2 h at 37 °C. Bacterial cell densities were measured by OD_600_. Cell cultures (1 ml) were collected by centrifugation and lysed in 10 μl of Laemmli buffer per 0.1 OD_600_ (Bacterial lysate). Secreted proteins were collected from the surface of the culture tube by addition of 10 μl of Laemmli buffer per 0.1 OD_600_, followed by incubation with shaking for 30 min at 37 °C (Secreted). Samples were analysed by SDS-PAGE and immunoblotting.

### RNA extraction and quantitative RT-PCR

RNA was extracted from bacterial strains grown in minimal medium pH 5.0 for 4 h using an RNAeasy mini kit (Qiagen) following the manufacturer’s instructions. Genomic DNA was digested with RNase-free DNase (Qiagen), following the manufacturer’s instructions. 400 ng of RNA was used to synthesize complementary DNA (QuantiTect RT kit, Qiagen) and 0.5 μl of cDNA was used for quantitative RT-PCR (reverse transcription polymerase chain reaction) using SybrGreen PCR master mix (Applied Biosystems). Data are represented as relative amounts of mRNA normalized to a 5S control. *steD* was amplified using the following primers: 5′-TCCTTCTGAACGGGGTAATG-3′ and 5′-TATGCCGCAC-CACTACTGAC-3′. 5S was amplified using the following primers: 5′-ACTAGCGCGGTGGTCCC-3′ and 5′-GCAGTTCCCTACTCTCGCATG-3′. quantitative RT-PCR was carried out on RNA samples without reverse transcription to check for lack of genomic DNA contamination.

### Immunoprecipitation

Bacterial strains were grown overnight in LB medium supplemented with appropriate antibiotics and subcultured into MgM-MES medium pH 5.0 for 5 h to induce SPI-2 gene expression. Cells were collected and re-suspended in 500 mM NaCl, 1 mM PMSF in PBS. Cells were lysed by son-ication, followed by addition of 0.5% Triton X-100 with vortexing and intact cells were removed by centrifugation (10 min, 16 000 **g**). Proteins were immunoprecipitated from the supernatant using anti-FLAG M2 affinity gel (Sigma) for 2 h at 4 °C. Samples were subsequently washed and analysed by SDS-PAGE and immunoblotting.

### Recombinant protein expression

*steD* was ligated into the pQlinkH vector with a 6HIS tag and *srcA* was ligated into the pQlinkN vector without a tag; both vectors were subsequently combined as described elsewhere [14]. The final vector was electroporated into *E. coli* BL21 and grown in Studier Auto Induction Medium in high density shaking cultures [15]. Cells were lysed by sonication in 20 mM phosphate buffer pH 8.0, containing 150 mM NaCl, 10 mM imidazole, 0.5 mM PMSF. After clarification by centrifugation, the lysate was incubated with nickel-nitri-lotriacetic acid (NiNTA) resin for 2 h at 4 °C with gentle rocking. The resin was extensively washed with 20 mM phosphate buffer pH 8.0, containing 150 mM NaCl and 20 mM imidazole, and the SteD-SrcA complex was eluted with 20 mM phosphate buffer pH 6.8, containing 150 mM NaCl, 500 mM imidazole and 1 mM DTT. The HIS tag was removed by incubation with tobacco etch virus protease at a ratio of 1 mg protease to 30 mg of the SteD-SrcA complex for 1 h at room temperature and overnight incubation at 4 °C then dialysed against 20 mM phosphate buffer pH 6.8, containing 150 mM NaCl, 5 % glycerol and 1 mM DTT. The untagged SteD-SrcA complex was purified by passing the dialysed sample through a second NiNTA column and concentrated using an Amico Ultra-15 centrifugal filter unit with Ultracel-10K membrane (Millipore). The concentrated SteD-SrcA complex was loaded onto a preparative Super-dex200 gel filtration column (GE Healthcare) pre-equili-brated in the dialysation buffer and eluted at a flowrate of 1 ml min^−1^. The protein samples were prepared for SDS-PAGE electrophoresis in Laemmli buffer without boiling to avoid aggregation induced by unfolding of hydrophobic regions [16].

### Mass spectrometry

Mass spectrometry analysis was carried out in the Laboratory of Mass Spectrometry, Institute of Biochemistry and Biophysics, Polish Academy of Sciences. Protein samples were excised from the SDS-PAGE gel, destained and subjected to a standard ‘in-gel digestion’ procedure during which proteins were reduced with 100 mM DTT (30 min at 56 °C), alkylated with iodoacetamide (45 min in darkroom at room temperature) and digested overnight with trypsin (sequencing Grade Modified Trypsin - Promega V5111). Resulting peptides were eluted from the gel with 0.1 % TFA, 2 % acetonitrile (ACN). Peptide mixtures were separated by liquid chromatography prior to molecular mass measurements on an Orbitrap Velos mass spectrometer (Thermo Electron, San Jose, CA). Peptide mixtures were applied to an RP-18 precolumn (nanoACQUITY Symmetry C18 - Waters 186003514) using water containing 0.1 % TFA as the mobile phase and then transferred to a nano-HPLC RP-18 column (nanoACQUITY BEH C18 - Waters 186003545) using an acetonitrile gradient (0–60 % ACN in 70 min) in the presence of 0.05 % formic acid with a flowrate of 150 nl min^−1^. The column outlet was directly coupled to the ion source of the spectrometer working in the regime of data dependent MS to MS/MS switch. A blank run ensuring lack of cross contamination from previous samples preceded each analysis. Acquired raw data were processed by Mascot Distiller followed by a database search with Mascot program (Matrix Science, London, UK) against SwissProt with taxonomy restriction to the *S. Typhimurium* database. Search parameters for precursor and product ions mass tolerance were 20 ppm and 0.4 Da, respectively, with trypsin specificity and the following variable modifications: cysteine carba-midomethylation and methionine oxidation. Peptides with a Mascot score exceeding the threshold value corresponding to <5 % expectation value, calculated by Mascot procedure, were considered to be positively identified.

### Size exclusion chromatography-multiangle light scattering (SEC-MALS)

The SteD-SrcA complex purified as described above was concentrated to 8 mg ml^−1^ and analysed by SEC-MALS by use of an in-line HPLC (Agilent Technologies 1260 Infinity), MALS system (Wyatt DAWN HELEOS II, and OPTI-LAB T-rEX) and analytical Superdex200 gel filtration column (GE Healthcare) in the dialysis buffer at a flowrate of 0.4 ml min^−1^. Astra chromatography software (Wyatt) was used for collecting and analysing data.

## Results And Discussion

### SrcA is required for Salmonella-induced reduction of surface mMHCII

**Fig. 1:**
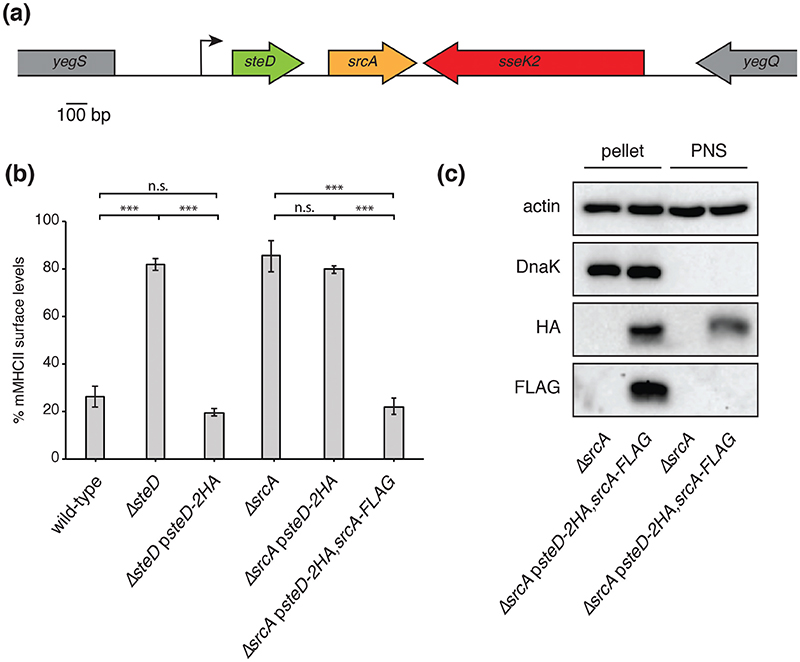
SrcA is required for Salmonella-induced reduction of surface mMHCII. (a) Chromosomal organization of *steD* and *srcA*, showing their genomic context and transcription start site. (b) Mel Juso cells were infected with indicated *Salmonella* strains for 20 h. Where indicated, *Salmonella* strains carried plasmid pWSK29 expressing SteD-2HA under its endogenous promoter, with or without SrcA-FLAG. mMHCII surface levels were measured by flow cytometry using mAB L243, which specifically recognizes mature HLA-DR. Infected cells were labelled with anti-CSA-1 antibody after fixation and permeabilization. The mMHCII surface levels are represented as a percentage of median fluorescence of infected cells over the median fluorescence of non-infected cells from the same sample. (Mean of three independent experiments done in duplicate±SD. Data were analysed by one-way ANOVA followed by Dunnett’s multiple comparison test, ***P<0.001.) (c) Mel Juso cells were infected with indicated *Salmonella* strains for 20 h. Cells were lysed in 0.1 % Triton X-100 and incubated on ice for 15 min with vortexing. The post-nuclear supernatant (PNS) was separated from the nuclear pellet and non-lysed *Salmonella* cells by centrifugation. The Western blot is representative of results from more than three independent experiments.

Analysis of *Salmonella* genome organization revealed that the gene encoding the previously characterized T3SS chap-erone SrcA [12] lies immediately downstream from the gene encoding the SPI-2 T3SS effector SteD, with 121 base pairs separating the two ORFs (Fig. 1a). Analysis of mRNA levels from *Salmonella* in macrophages indicates that both genes have the same transcriptional start site and that transcription stops after *srcA* [17]. Therefore, *steD* and *srcA* are part of the same operon. This operon is located, along with *sseK2*, between *yegS* and *yegQ* (Fig. 1a). Examination of the *E. coli* genome using the EcoGene database, revealed that both *yegS* and *yegQ* are present in *E. coli*, whereas *srcA, steD* and *sseK2* are not. SteD is one of a subgroup of core SPI-2 T3SS effectors, whose genes are present in almost all sero-vars of *Salmonella* [8]. The genomic sequence of the entire operon including *steD* and *srcA* from *Salmonella enterica* serovar Typhimurium was used to search the NCBI database with BLAST. Of 564 *Salmonella* genome sequences, both *srcA* and *steD* are present in 562, representing 82 different serovars, and in the remaining two strains neither gene is present. Together, this indicates that *steD* and *srcA* were co-acquired by horizontal transfer. SrcA, which has structural similarity to the *E. coli* chaperone CesT, was characterized as a *Salmonella* chaperone for the SPI-2 T3SS effectors PipB2 and SseL [12]. T3SS class IA chaperones are often encoded in the vicinity of their cognate effector genes [3–5]. To establish if SrcA is involved in SteD function, we first tested if SrcA is also required for the reduction of mMHCII surface levels. For this we created an *srcA* in-frame deletion mutation in *Salmonella* strain 14028s and used the mutant strain to infect Mel Juso cells, a melanoma cell line commonly used to study MHCII trafficking and presentation [10, 11, 18]. mMHCII surface levels were detected with the monoclonal antibody L243, which recognizes mature HLA-DR, one of the three human MHCII isotypes [19]. As expected, wild-type *Salmonella* caused a decrease in surface levels of mMHCII compared to non-infected cells (Figs 1b and S1). As previously reported [11], this effect was abrogated by infection with the *DsteD* strain and restored by a mutant strain carrying a plasmid-encoding SteD-2HA under the control of its endogenous promoter (p*steD-2HA*), (Figs 1b and S1). A complete prevention in the reduction in mMHCII surface levels also occurred after infection with the *DsrcA* strain (Figs 1b and S1). As *steD* and *srcA* are adjacent in the chromosome it was important to verify that the phenotype of the D*srcA* strain was not the result of an indirect effect of the *srcA* deletion on *steD* function. The plasmid containing *steD-2HA* failed to complement the D*srcA* mutant. However, a plasmid carrying both *steD-2HA* and *srcA-FLAG (psteD-2HA, srcA-FLAG*), fully complemented the D*srcA* phenotype (Figs 1b and S1). Therefore, SrcA is also required for the reduction in mMHCII surface levels.

To determine if SrcA is translocated into host cells or is confined within *Salmonella* cells, we fractionated Mel Juso cells after infection with *Salmonella* carrying p*steD-2HA, srcA-FLAG.* After Mel Juso cell lysis, *Salmonella* cells along with host cell nuclei were removed by centrifugation to leave a post-nuclear supernatant (PNS), containing host cell and translocated proteins. DnaK, a cytoplasmic *Salmonella* protein, was detected in the pellet but not in the PNS, indicating negligible bacterial contamination of the PNS. As expected, SteD-2HA was present both in the pellet and PNS, indicating translocation of the protein. However, SrcA-FLAG was only detected in the pellet fraction (Fig. 1c), indicating that it is not translocated. Since SrcA-FLAG was functional in reducing mMHCII surface levels (Fig. 1b), we conclude that SrcA functions within *Salmonella*.

### SrcA is required for secretion and translocation of SteD

**Fig. 2:**
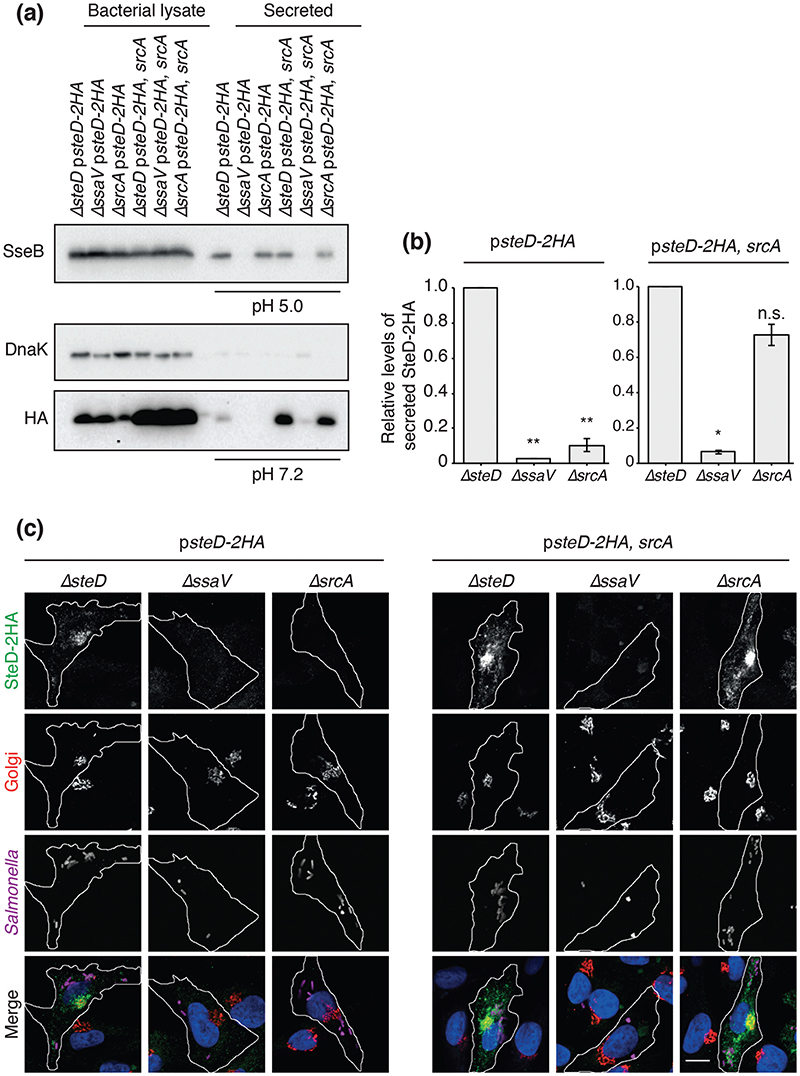
SrcA is required for secretion and translocation of SteD into host cells. (a) The indicated *Salmonella* strains were grown in minimal medium pH 5.0 for 4h then washed and transferred to minimal medium pH 7.2 for 2 h. All *Salmonella* strains carried plasmid pWSK29 expressing SteD-2HA under its endogenous promoter, with or without SrcA. Bacterial lysate proteins and secreted proteins at either pH 5.0 or pH 7.2 were examined by immunoblotting with antibodies against DnaK, the HA epitope or SseB. (b) Levels of secreted SteD-2HA were calculated by densitometry from immunoblots against HA using Image Lab software. Secreted protein levels from the *ΔssaV* and *ΔsrcA* strains are shown in relation to the *ΔsteD* strain. Mean of three independent experiments ±SD. The log_10_ of the ratios of secreted protein levels from the *ΔssaV* or *ΔsrcA* over that of the *ΔsteD* strain were analysed by one sample t-test, **P<0.01, *P<0.05. (c) Representative confocal immunofluorescence microscopy images of SteD-HA localization in Mel Juso cells at 20 h p.i. with the indicated *Salmonella* strains. Cells were fixed and processed for immunofluorescence microscopy by labelling for SteD-2HA (anti-HA antibody, green), the Golgi network (anti-GM130 antibody, red), *Salmonella* cells (anti-CSA-1 antibody, magenta) and DNA (DAPI, blue). Scale bar, -10 μm.

T3SS chaperones are either required for or regulate effector translocation into host cells. Therefore, we tested if SrcA is required for translocation of SteD from *Salmonella*. The physiological signal for translocation of SPI-2 T3SS effectors is the near-neutral pH of the host cell cytoplasm, which is sensed by the T3SS machinery after its assembly in response to the acidic pH of the vacuole lumen [13]. This can be mimicked *in vitro* by shifting the ambient pH of minimal medium from 5.0 to 7.2, which leads to derepression of SPI-2 T3SS effector secretion [13]. We used this to test the requirement of SrcA for secretion of SteD-2HA expressed from p*steD-2HA*. DnaK was used to check that the secreted fraction was not contaminated with intact or lysed bacterial cells. To verify that deletion of *srcA* did not impede general secretion from the SPI-2 T3SS we examined the secreted fraction before the pH shift to pH 7.2 for the presence of the SPI-2 translocon component SseB. SseB was not detected in the secreted fraction of strains carrying a mutation in *ssaV*, which prevents any secretion from the SPI-2 T3SS. However, SseB was detected in the secreted fraction of the Δ*srcA* strain (Fig. 2a). As expected, SteD-2HA was detected after the pH shift in the secreted fraction from the Δ*steD* strain but not from the Δ*ssaV* mutant (Fig. 2a, b). Deletion of *srcA* did not prevent the production of SteD-2HA in *Salmonella* cells but did prevent its secretion. The SteD secretion defect in D*srcA* was complemented by the presence of *srcA* on a plasmid (p*steD-2HA*, *srcA*) (Fig. 2a, b). The levels of both intrabacterial and secreted SteD-2HA were noticeably higher in the presence of plasmid-encoded SrcA, whereas deletion of *srcA* resulted in a decrease in intrabacterial SteD-2HA levels (Fig. 2a). Therefore, SrcA is required for SteD secretion and the amount of SteD in *Salmonella* cells is related to the amount of SrcA.

To test if SrcA is required for the translocation of SteD from intracellular bacteria we tested for the presence of SteD-2HA in Mel Juso cells by immunofluorescence microscopy after infection with *Salmonella* strains carrying p*steD-2HA*. SteD localizes mainly at the Golgi network of host cells following its translocation [11]. In agreement with the *in vitro* secretion assay, SteD was not detected in Mel Juso cells after infection with the D*srcA* mutant or after infection with the D*ssaV* strain (Fig. 2c). On the other hand, the presence of *srcA* on the plasmid rescued the translocation of SteD into Mel Juso cells (Fig. 2c). These results suggest that SrcA is a chaperone for SteD and explain its requirement for reducing mMHCII surface levels in antigen-presenting cells.

### SrcA is not required for the secretion of the SPI-2 T3SS effectors SseL and PipB2

**Fig. 3:**
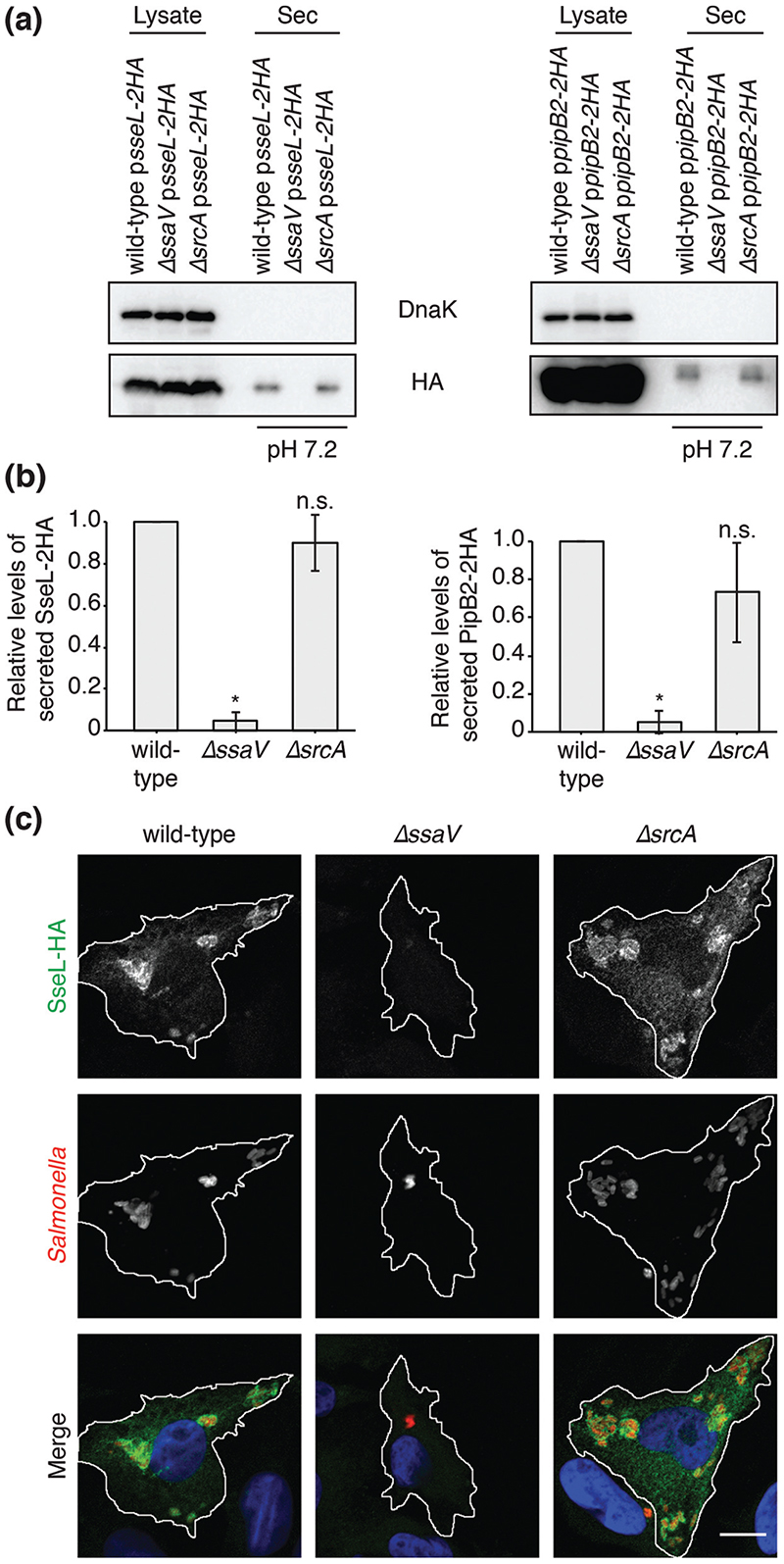
SrcA is not required for the secretion of the SPI-2 T3SS effectors SseL and PipB2. (a) The indicated *Salmonella* strains were grown in minimal medium pH 5.0 for 4 h, then washed and transferred to minimal medium pH 7.2 for 2 h. *Salmonella* strains carried plasmid pWSK29 expressing either SseL-2HA under its endogenous promoter or PipB2-2HA under the SseA promoter. Bacterial lysate proteins and secreted (Sec) proteins at pH 7.2 were examined by immunoblotting with antibodies against DnaK or the HA epitope. (b) Levels of secreted HA-tagged protein were calculated by densitometry from immunoblots against HA using Image Lab software. Protein levels in *ΔssaV* and *ΔsrcA* strains are shown in relation to the wild-type strain. Mean of three independent experiments ±SD. The log_10_ of the ratios of secreted protein levels from the *ΔssaV* or *ΔsrcA* over that of the *ΔsteD* strain were analysed by one sample t-test, *P<0.05. (c) Representative confo-cal immunofluorescence microscopy images of SseL localization in Mel Juso cells at 20 h p.i. with the indicated *Salmonella* strains. Cells were fixed and processed for immunofluorescence microscopy by labelling for SseL (anti-HA antibody, green), *Salmonella* cells (anti-CSA-1 antibody, red) and DNA (DAPI, blue). Scale bar, - 10 μm.

SrcA was first characterized as a chaperone for the SPI-2 T3SS effectors SseL and PipB2 [12]. However, the position of *srcA* and *steD* within the same operon (Fig. 1a) suggests that SrcA might be a specific chaperone for SteD. To test if SrcA is also required for the secretion of SseL and PipB2, we used the *in vitro* pH shift secretion assay described above. As expected, SseL-2HA and PipB2-2HA were secreted after expression from a plasmid in wild-type *Salmonella*, but were not secreted from the D*ssaV* strain (Fig. 3a, b). In contrast to SteD, similar amounts of secreted SseL and PipB2 were detected in the presence or absence of SrcA (Fig. 3a, b). This is in contrast to previously published results, in which secretion of SseL and PipB2 was not detected in the absence of SrcA [12]. This discrepancy could be due to methodological differences in the *in vitro* secretion assays. Cooper *et al*. tested for secretion at pH 5.8 and collected secreted proteins by trichloro-acetic acid precipitation of culture supernatants [12], whereas our assay involved collection of secreted proteins from the surface of the culture tube after shifting the ambient pH from 5.0 to 7.2, to mimic sensing of the neutral pH of the host cell cytoplasm [13]. Translocated SseL-2HA was also readily detected in Mel Juso cells after infection with the wild-type or D*srcA* strain (Fig. 3c). Although we cannot exclude the possibility that SrcA is a chaperone for other effectors, our results show that it is not required for the secretion of SseL or PipB2 in conditions that reflect physiological activation of SPI-2 T3SS effector secretion.

### SrcA maintains SteD stability

**Fig. 4:**
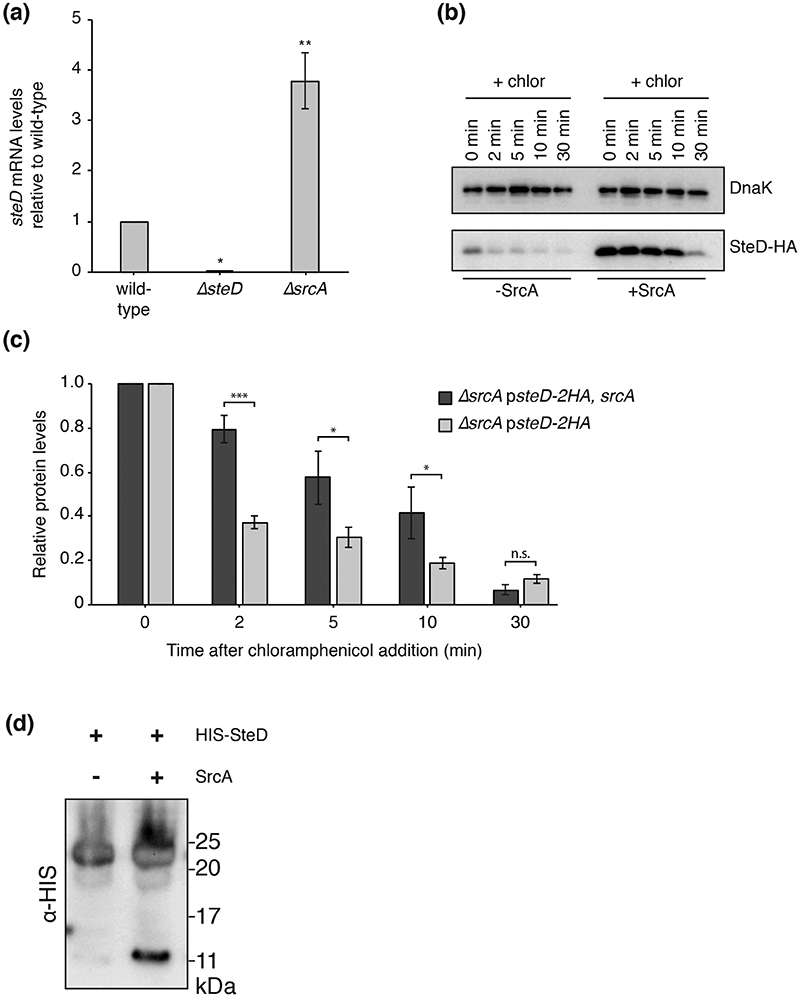
SrcA maintains SteD stability. (a) quantitative RT-PCR of *steD* mRNA levels compared to those in wild-type *Salmonella*. Mean of three independent experiments ±SD. The log_10_ of the ratios of mRNA levels in the *ΔsteD* or *ΔsrcA* strains over those in the wild-type strain were analysed by one sample t-test, **P<0.01, *P<0.05. (b) *ΔsrcA Salmonella* strains carrying plasmid pWSK29 expressing SteD-2HA under its endogenous promoter with or without SrcA were grown in minimal medium pH 5.0 for 4 h. Chloramphenicol (chlor; 50 ug ml^−1^) was added at 0 min. At indicated times cell cultures (1 ml) were collected by centrifugation and lysed in 10 μl of Laemmli buffer per 0.1 OD_600_. Samples were examined by immunoblotting with antibodies against the HA epitope or DnaK. (c) Levels of cellular SteD-2HA were calculated by densitometry from immunoblots against HA using Image Lab software. Protein levels at indicated time points post chloramphenicol addition are shown in relation to those immediately before chloramphenicol addition. Mean of three independent experiments ±SD. Data were analysed by t-test, ***P<0.001, *P<0.05. (d) HIS-SteD was expressed in *E. coli* strain BL21 under the lac promoter in the presence or absence of ScrA and the protein complex was purified to homogeneity from the soluble fraction. Immunoblotting using an antibody against the HIS epitope was used to visualize SteD.

T3SS effectors are produced and stored in the bacterial cytoplasm before their secretion, which allows rapid secretion upon the correct physiological trigger [4, 20]. Class I chap-erones can maintain the stability of the effector in the bacterial cytoplasm [5, 6]. The absence of SrcA resulted in lower amounts of plasmid-expressed SteD in *Salmonella* cells (Fig. 2a), which could be due to either a reduction in SteD expression or an increase in its degradation. Measurement of the *steD* mRNA levels by quantitative RT-PCR showed that they were greater in the absence of SrcA, indicating that the reduction in protein was not due to a decrease in expression of SteD (Fig. 4a). To test the stability of SteD in the absence of SrcA, *de novo* translation was blocked using chloramphenicol and the rate of degradation of SteD-2HA was measured by determining protein levels over time by Western blot (Fig. 4b). The initial levels of SteD were reduced in the absence of SrcA, so the rate of degradation was calculated as the relative amount of protein compared to initial levels (Fig. 4c). In the absence of SrcA, there was a significant reduction in levels of SteD within 2 min of chlor-amphenicol addition. There was strikingly less SteD degradation in the presence of SrcA (*ΔsrcA* p*steD-2HA*, *srcA*). Therefore, SrcA helps to maintain the stability of SteD in *Salmonella* cells.

We next purified SteD in the presence or absence of SrcA. Recombinant HIS-tagged SteD was largely insoluble when expressed in *E. coli*. The soluble HIS-SteD fraction was recovered from the bacterial lysate using NiNTA agarose. After elution from NiNTA agarose, recombinant HIS-SteD migrated through an SDS polyacrylamide gel with an apparent mass between 20 and 25 kDa (Fig. 4d), which is closer to that expected of a HIS-SteD dimer (28 kDa). The presence of this higher molecular weight species suggests that SteD was not fully solubilized at room temperature in Laemmli buffer and self-aggregated into a complex of at least two SteD molecules. When HIS-SteD was co-expressed with SrcA, some HIS-SteD molecules subsequently migrated through the SDS polyacrylamide gel with a size closer to that expected of a monomer (14kDa; Fig. 4d). The difference between the expected and apparent mass of both dimer and monomer might be due to the gel-shifting phenomenon, in which positively charged residues (for example, in the linker region between the HIS tag and SteD) bind additional SDS molecules and thus increase migration through the gel [21]. Together these results suggest that an interaction with SrcA partially prevents SteD self-aggregation, which could help maintain SteD stability.

As there was some SteD-2HA still present in *Salmonella* cells in the absence of SrcA (Fig. 2a), this suggests that SrcA not only maintains SteD stability but also enables its secretion. Interaction with a chaperone can lead to partial unfolding of the effector, maintaining it in a secretion-competent state [22, 23] and it is possible that in preventing SteD self-aggregation the interaction with SrcA allows SteD secretion. It has also been shown that SrcA interacts with SsaN, an ATPase that is part of the secretion machinery [12, 24], so the interaction between SrcA and SsaN could be required for the recognition and entry of SteD into the SPI-2 T3SS.

### SrcA interacts directly with SteD

**Fig. 5:**
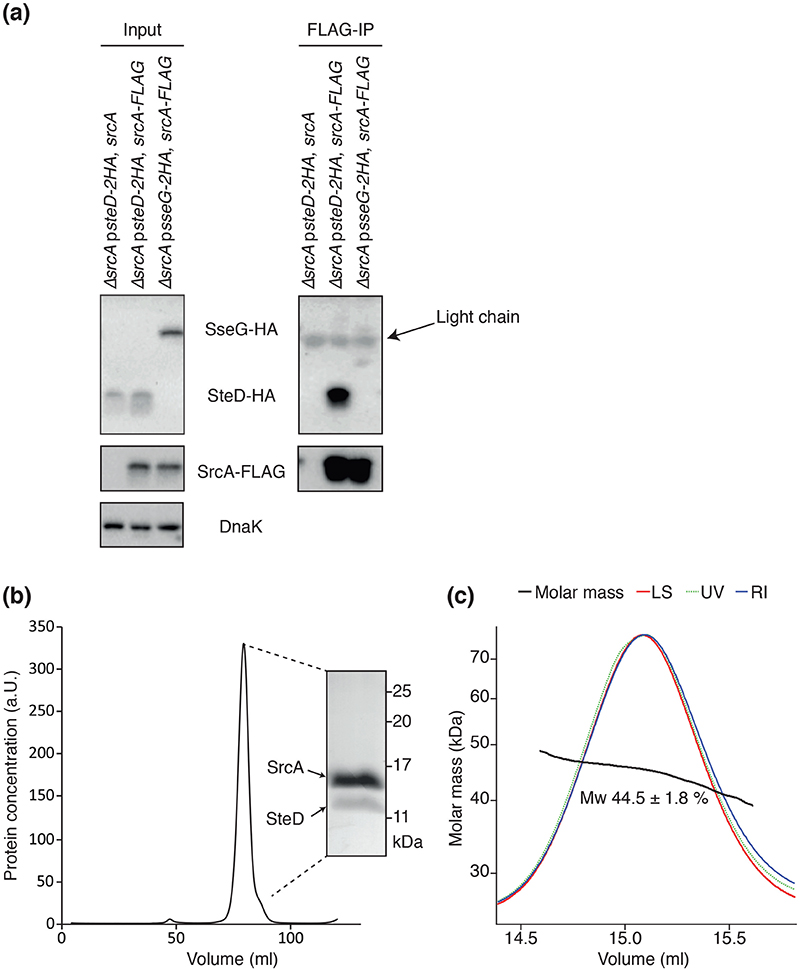
SrcA interacts directly with SteD (a), Indicated *Salmonella* strains were grown in minimal medium pH 5.0 for 5h. Cells were then pelleted and frozen overnight. Cells were resuspended in lysate buffer (500 mM NaCl, 1 mM PMSF in PBS) and lysed by sonication, followed by addition of Triton X-100. Proteins were immunoprecipitated with anti-FLAG M2 affinity gel and samples were analysed by immunoblotting with antibodies against HA, FLAG and DnaK. Light chain indicates the light chain of the anti-FLAG antibody in the affinity gel, which is also detected by the secondary antibody in the Western blot. (b) A complex of SteD and SrcA was expressed in *E. coli* from a single plasmid under the lac promotor and purified to homogeneity on NiNTA beads. The size of the complex was examined using size exclusion chromatography. The obtained peak was analysed using SDS-PAGE and subsequent mass spectrometry of the protein bands. (c) Elution profile of the SteD-SrcA complex examined by size exclusion chromatography-multiangle light scattering (SEC-MALS). The traces of MALS calculated molar mass (black, molar mass), light scattering (red, LS), UV absorbance (dotted green, UV) and differential refractive index (blue, RI) are shown. Calculated weight-averaged molar mass (Mw) is indicated below the black molar mass curve.

To test if SrcA and SteD interact in *Salmonella*, cells carrying p*steD-2HA*, *srcA-FLAG* were lysed and SrcA-FLAG was subjected to pull-down with an anti-FLAG antibody and tested for co-immunoprecipitation of SteD-2HA. As a control, we used 2HA-tagged SseG, another integral membrane effector of the SPI-2 T3SS. No interaction between SrcA-FLAG and SseG-2HA was detected (Fig. 5a). SteD-2HA was efficiently co-immunoprecipitated with SrcA-FLAG but not from the sample where untagged SrcA was used, showing that the co-immunoprecipitation of SteD-2HA was specific (Fig. 5a).

To test if SteD and SrcA interact directly, we checked for complex formation after co-expression of both proteins in *E. coli*. HIS-SteD and SrcA were expressed from the same plasmid and purified to homogeneity using two cycles of NiNTA affinity chromatography followed by size exclusion using a S200 Superdex column. After cleavage of the HIS-tag, the proteins formed a soluble complex, which co-eluted from the size exclusion column as a single peak (Fig. 5b). The proteins collected from this peak migrated as two species on an SDS polyacrylamide gel. These proteins were cut from the gel and were identified as SrcA and SteD by mass spectrometry analysis. The stronger intensity of the SrcA band (Fig. 5b) suggested more SrcA than SteD molecules in the complex. To determine the stoichiometry of the complex, we used size exclusion chromatography-multiangle light scattering (SEC-MALS). The molar mass of the SteD-SrcA complex was calculated to be 44.5 kDa, which, given the predicted molecular masses of monomeric, untagged SrcA (16.1 kDa) and SteD (11.4 kDa), shows that the stoichiometry of the complex is 2 SrcA molecules:1 SteD molecule (Fig. 5c). This is in agreement with structural data of SrcA, showing that it forms a dimer [12]. Additionally, several other T3SS effectors have been shown to interact with chaper-ones with a dimer interface [23, 25]. Further work is needed to confirm the exact interface of the interaction and how the interaction with SrcA affects the structure and stability of SteD. One possibility is that SrcA interacts with the hydrophobic transmembrane regions of SteD to maintain its solubility. However, several T3SS effectors, including the *E. coli* integral membrane effector, Tir, interact with their cognate chaperones via their N-terminal regions [5, 26].

These results show that SrcA and SteD interact both *in vitro* and in the *Salmonella* cytoplasm. Together with the findings that SrcA is not secreted from *Salmonella* cells but is required for SteD stability and secretion, our results show that SrcA is a chaperone for SteD.

## Supplementary Material

Supplementary Material
