# Supplementary material for: SrcA is a chaperone for the Salmonella SPI-2 type three secretion system effector SteD
Source: Microbiology (Reading). Author manuscript; Available in PMC 2023 Sep 27. (PMC7614968; doi:10.1099/mic.0.000732)
Supplement: Supplementary Material [file EMS185005-supplement-Supplementary_Material.pdf]

Fig. S1

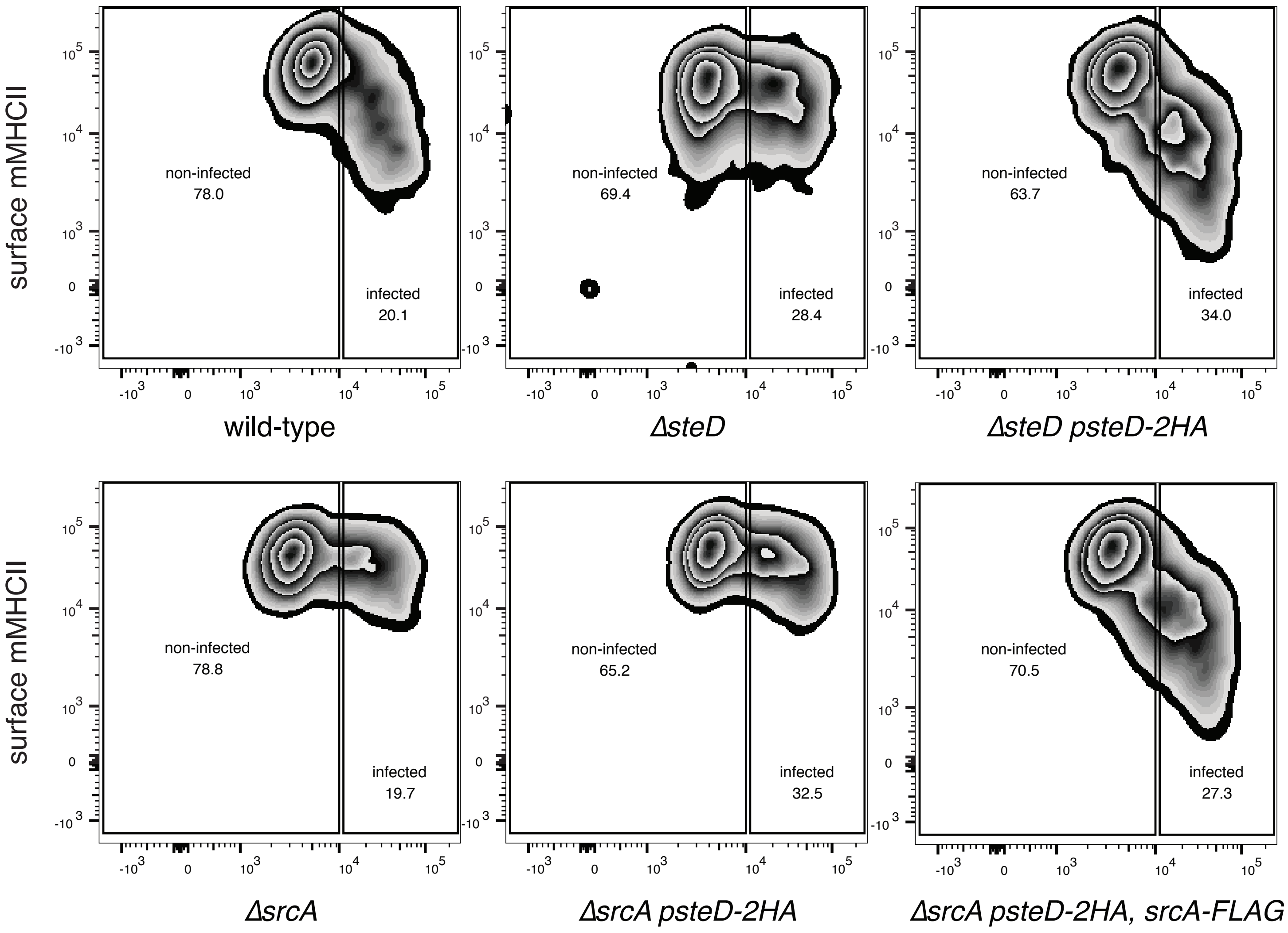

Fig. S1
Representative flow cytometry plots showing surface levels of mMHCII in infected cells compared to non-infected cells. Numbers shown are percentage of total cells in each gate. Y axis: Surface mMHCII L243 antibody; X axis anti-*Salmonella* CSA-1 antibody.

Table S 1.

***S.* Typhimurium strains used in this study**

Name	Description	Reference
wild-type	14028s *S.* Typhimurium wild-type	ATCC
ΔssaV	*ΔssaV* ::km	Beuzon et al., 1999
ΔsteD	*ΔsteD* ::km	Bayer-Santos et al., 2016
ΔsrcA	*ΔsrcA* ::cm	This study

Abbreviations: ATCC - American Type Culture Collection, km - kanamycin resistance, cm - chloramphenicol resistnace (Datsenko et al., 2000)

Table S 2.

Plasmids used in this study

Name	Description	Reference
p*steD-2HA*	pWSK29 containing C-terminal 2HA-tagged *steD* with endogenous promoter	Bayer-Santos et al., 2016
p*steD-2HA, srcA*	pWSK29 containing C-terminal 2HA-tagged *steD* and *srcA* including intergenic region with endogenous promoter	This study
p*steD-2HA, srcA-FLAG*	pWSK29 containing C-terminal 2HA-tagged *steD* and C-terminal FLAG-tagged *srcA* including intergenic region with endogenous promoter	This study
p*sseL-2HA*	pWSK29 containing C-terminal 2HA-tagged *sseL* with endogenous promoter	Rytkonen et al., 2007
p*pipB2-2HA*	pWSK29 containing C-terminal 2HA-tagged *pipB2* with *sseA* promoter	This study
p*sseG-2HA, srcA-FLAG*	pWSK29 containing C-terminal 2HA-tagged *sseG* and C-terminal FLAG-tagged *srcA* with *sseA* promoter	This study
pQlink *HIS-steD*	pQlink containing N-terminal 6HIS-tagged *steD* with lacZ promoter	This study
pQlink *HIS-steD, srcA*	pQlink containing N-terminal 6HIS-tagged *steD* and untagged SrcA with lacZ promoter	This study

Table S 3.

Primary antibodies used in this study

Specificity	Clone	Use	Source	Dilution
mMHCII	Mouse monoclonal L243	FACS	Sigma-Aldrich	1 in 300
CSA-1	Goat polyclonal	FACS/IF	KPL	FACS 1 in 500/ IF 1 in 200
actin	Rabbit polyclonal	WB	Sigma-Aldrich	1 in 2000
DnaK	Mouse monoclonal 8E2/2	WB	Enzo	1 in 2000
HA.11	Mouse monoclonal 16B12	WB	Covance	1 in 1000
FLAG	Rabbit polyclonal	WB	Sigma-Aldrich	1 in 2000
SseB	Rabbit polyclonal	WB	Beuzon et al.	1 in 5000
HIS	Rabbit polyclonal	WB	Abcam	1 in 5000
HA	Rat monoclonal 3F10	WB/IF	Roche	WB 1 in 2000/ IF 1 in 200
GM130	Mouse monoclonal 35	IF	BD Biosciences	1 in 400

Abbreviations: IF - immunofluorescence, WB - western blot

Supplemental references

Beuzón CR, Banks G, Deiwick J, Hensel M, Holden DW. pH-dependent secretion of SseB, a product of the SPI-2 type III secretion system of Salmonella typhimurium. *Mol Microbiol* 1999;33:806–816.

Datsenko KA, Wanner BL. One-step inactivation of chromosomal genes in Escherichia coli K-12 using PCR products. *Proc Natl Acad Sci* 2000;97:6640–6645.

Rytkonen A, Poh J, Garmendia J, Boyle C, Thompson A, et al. SseL, a Salmonella deubiquitinase required for macrophage killing and virulence. *Proc Natl Acad Sci* 2007;104:3502–3507.
